# Author Correction: Environment-responsive nanophores for therapy and treatment monitoring via molecular MRI quenching

**DOI:** 10.1038/s41467-019-09887-3

**Published:** 2019-04-18

**Authors:** Charalambos Kaittanis, Travis M. Shaffer, Anuja Ogirala, Santimukul Santra, J. Manuel Perez, Gabriela Chiosis, Yueming Li, Lee Josephson, Jan Grimm

**Affiliations:** 1 Molecular Pharmacology and Chemistry Program, Memorial Sloan Kettering Cancer Center, 1275 York Avenue, New York, New York 10065 USA; 2 Department of Chemistry, Hunter College of the City University of New York, Graduate Center, New York, New York 10065 USA; 3 Department of Chemistry, Pittsburg State University, 1701 S Broadway Street, Pittsburg, Kansas 66762 USA; 4 NanoScience Technology Center, University of Central Florida, 12424 Research Parkway, Suite 400, Orlando, Florida 32826 USA; 5 Center for Advanced Medical Imaging Sciences, Massachusetts General Hospital, Building 149, 13th Street, Charlestown, Massachusetts 02129 USA

Correction to: *Nature Communications* 10.1038/ncomms4384, published online 04 March 2014

This Article contains an error in Fig. 6. In panel b, the left-hand image is mistakenly described as showing fluorescence before treatment, while it in fact shows the same white light image as the right-hand panel without fluorescent overlay to better visualize the tumour location. A correct version of Fig. 6b is presented below as Figure 1. The error has not been corrected in the original version of the Article.

**Figure 1:**
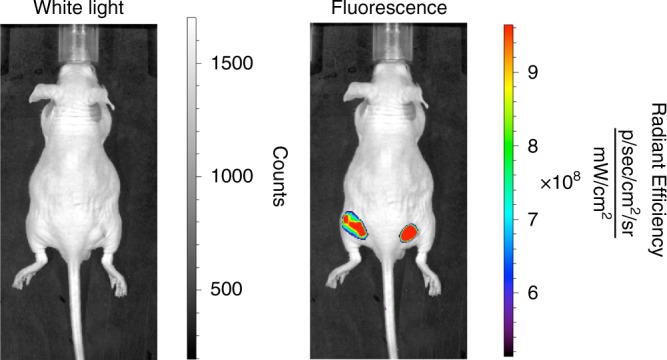
Fig. 1

